# Association of Park Renovation With Park Use in New York City

**DOI:** 10.1001/jamanetworkopen.2024.1429

**Published:** 2024-04-10

**Authors:** Hanish P. Kodali, Katarzyna E. Wyka, Sergio A. Costa, Kelly R. Evenson, Lorna E. Thorpe, Terry T.-K. Huang

**Affiliations:** 1 Center for Systems and Community Design, Graduate School of Public Health and Health Policy, City University of New York (CUNY), New York, New York; 2 NYU-CUNY Prevention Research Center, New York, New York; 3 Department of Epidemiology, Gillings School of Global Public Health, University of North Carolina–Chapel Hill; 4 Department of Population Health, Grossman School of Medicine, New York University (NYU), New York, New York

## Abstract

**Importance:**

Equity-driven citywide park redesign and renovation, such as the Community Parks Initiative (CPI), has the potential to increase park use and opportunities for physical activity in underserved communities.

**Objective:**

To evaluate changes in patterns of park use following park redesign and renovation in low-income New York City (NYC) neighborhoods.

**Design, Setting, and Participants:**

The Physical Activity and Redesigned Community Spaces study was a prospective quality improvement preintervention-postintervention study design with matched control parks. Thirty-three intervention and 21 control neighborhood parks were selected based on specific criteria related to poverty rates, population growth, and population density in park neighborhoods and not having received more than $250 000 in investment in the past 2 decades. Data were collected at baseline (prerenovation) and 2 follow-up points (3 months and 1 year post renovation) between June 5 and December 4 from 2016 to 2022. Participants were individuals observed as users of study parks.

**Intervention:**

The CPI, which involved the redesign and renovation of neighborhood parks by the municipal government of New York City.

**Main Outcomes and Measures:**

Main outcomes encompassed park use and physical activity levels assessed using the well-validated System for Observing Play and Recreation in Communities. Park use was quantified by total number of park users, categorized by age group (≤20 years vs ≥21 years), sex, and physical activity level (sitting or standing vs walking or vigorous activity). Changes in outcomes between groups were compared via the generalized estimation equation.

**Results:**

A total of 28 322 park users were observed across 1458 scans. At baseline, 6343 of 10 633 users (59.7%) were 20 years or younger, 4927 of 10 632 (46.3%) were female and 5705 (53.7%) were male, and 4641 of 10 605 (43.8%) were sitting or standing. Intervention parks showed more net park users compared with control parks from baseline to the final follow-up (difference-in-difference relative rate ratio, 1.69 [95% CI, 1.22-2.35] users/scan; *P* = .002). The association was driven by a significant increase in adult users at intervention parks and overall decrease in all users at control parks. Park users engaging in sitting or standing at intervention parks increased (difference, 4.68 [95% CI, 1.71-7.62] users/scan; *P* = .002) and park users engaging in walking or vigorous physical activity at control parks decreased (difference, −7.30 [95% CI, −10.80 to −4.26] users/scan; *P* < .001) over time.

**Conclusions and Relevance:**

In this quality improvement study, park redesign and renovation were positively associated with park use in low-income neighborhoods. However, park renovations may need to be accompanied by other programmatic strategies to increase physical activity.

## Key Points

**Question:**

Is the Community Parks Initiative (CPI), a city-led park redesign and renovation initiative in New York City, associated with park use?

**Findings:**

In this quality improvement study, the CPI was associated with a significant net increase in park use at intervention compared with control parks over time, particularly among adults.

**Meaning:**

These findings suggest that improving the quality of parks could enhance park use in low-income communities.

## Teaser

This quality improvement study evaluates the association of the Community Parks Initiative (CPI) with changes in park use and level of physical activity in parks in New York City.

## Introduction

More than two-thirds of the world’s population is projected to reside in cities by 2050, which may further exacerbate health inequities among residents of low-income neighborhoods.^1^ To mitigate this, the World Health Organization emphasizes strategies aimed at planning, building, and maintaining cities to enhance community well-being. Among the different structures of the built environment, neighborhood parks have received increasing attention in urban planning and public health.^2,3,4^ Parks have the potential to provide opportunities for physical activity, mitigate heat and pollution, lower stress, and improve mental and social well-being, all of which can decrease the prevalence of chronic diseases.^2,5^

In a recent review encompassing 17 studies,^6^ only 9 focused on assessing the effects of park or playground renovations on park use and physical activity in the US. Among these, only 5 studies included control parks that did not receive renovation or construction. Furthermore, just 1 study included more than 5 parks and showed a substantial rise in park utilization and physical activity from baseline to the 12-month follow-up.^7^ However, due to the scarcity of studies and small sample sizes, drawing broad conclusions or estimates remains challenging.^6,8,9^ Studies have also shown mixed results regarding age and sex patterns of park use.^10,11^

Parks in underserved areas with higher proportions of Black and Latino residents with low income historically receive less funding and have fewer physical activity resources than those in predominantly White and wealthier neighborhoods across the US.^12,13^ In addition, residents in lower-income areas have been shown to engage in less physical activity than those in higher-income areas.^14^ In New York City (NYC), despite the ubiquity of parks, disparities in park quality and use persist.^15^

To address these disparities, the NYC Department of Parks & Recreation (NYC Parks) launched the Community Parks Initiative (CPI) in 2014, a $318 million equity-based redesign and renovation of 67 neighborhood parks.^16^ Selected parks were situated in densely populated and growing neighborhoods with higher-than-average concentrations of inhabitants living below the federal poverty line. The CPI enhanced the quality of parks by improving aesthetics, vegetation, seating areas, accessibility (eg, lowered fences), children’s play equipment, and exercise and sports facilities. The CPI represents a policy to address environmental and social justice, and in light of the potential role of parks in health, its implementation could also contribute to health equity. As the CPI rolled out in waves, it provided a unique opportunity to examine the effects of park redesign and renovation on park use and physical activity on an unprecedented scale. Thus, we evaluated the association of the CPI from baseline to 1 year post park renovation (or approximately 3 years post baseline) on changes in park use and level of physical activity in parks.

## Methods

### Design

The Physical Activity and Redesigned Community Spaces (PARCS) study used a prospective quality improvement preintervention-postintervention design with matched control parks.^17^ This study conformed to the Standards for Quality Improvement Reporting Excellence (SQUIRE), version 2.0, reporting guideline for quality improvement studies. This study, based on park audits only, was deemed by the City University of New York Institutional Review Board as research not involving human participants and therefore did not require informed consent. However, the larger PARCS study, in which the current study was embedded, was approved by the City University of New York Institutional Review Board.

### Intervention

The CPI is the first large-scale equity-based redesign and renovation project implemented by NYC Parks.^18^ To be included, parks needed to meet 2 of 3 selection criteria in each park neighborhood: high level of poverty (≥20% population below the federal poverty line), high population growth (25% from 2000 to 2010), and high population density (≥110 people/acre). In addition, to be eligible, selected parks must not have received more than $250 000 in investment in the prior 2 decades.

During the initial design phase, the city consulted a wide range of residents, including vulnerable populations. Based on community inputs and site conditions, innovative park designs were generated. This process aimed to make the parks more accessible, multipurpose, and able to serve different demographic populations (eg, older people in addition to children).^19^ Common features of designs included adding more seating and shaded areas, planting more trees and vegetation, restoring lawns, renovating ball courts and playground equipment, and improving the aesthetics of the spaces. Parks were closed for construction for 1 to 2 years. Construction took place in waves from 2017 to 2021.

### Study Setting

The present study included 33 intervention parks and 21 sociodemographically matched control parks throughout NYC.^17^ Both intervention and control parks were on the initial eligibility list for CPI, but control sites were not scheduled for renovation during the study period. In addition, control parks were selected based on frequency matches (±6%) on key aggregated sociodemographic characteristics of residents (eg, race and ethnicity, percentage of adults, poverty rate) living within the 0.3-mile radius buffer of each park.^19^ In these neighborhoods, more than 25% of the residents were Black and nearly 40% were Latino.^17^ Approximately 25% of the residents were younger than 18 years, and 30% of the households lived below the poverty line.^17^ Study parks were small neighborhood spaces, averaging 1 acre in size.

### Data Collection

Park use was captured using the validated and reliable System for Observing Play and Recreation in Communities (SOPARC), recording total number of park users and their characteristics and behaviors.^20,21^ Data were collected across 3 phases: baseline (wave 1, or before renovation), approximately 3 months post renovation or 2 years post baseline (wave 2), and 1 year post renovation or 3 years post baseline (wave 3) (eFigure 1 in Supplement 1). Observations were conducted from June 5 to December 4 between 2016 and 2022. All baseline data, 81% of wave 2 data, and 67% of wave 3 data were collected prior to the onset of the COVID-19 pandemic in 2020.

Parks were mapped and divided into zones for data collection and audited for at least 3 days (mean, 4.6 [range, 3-7] days; 2 scans/d), including weekdays and weekends. Trained project coordinators scanned each zone and captured park user data. Intervention sites had 1 to 9 zones at wave 1 and 3 to 19 during waves 2 and 3, while control sites had 2 to 8 zones at all points. SOPARC recorded 3 attributes of each park user: age group (child 12 years or younger, teen aged 13 to 20 years, adult aged 21 to 59 years, or senior 60 years or older), sex (male or female), and physical activity (sitting, standing, walking, and vigorous). Race and ethnicity data were not collected because they could not be accurately coded in the park audit.

### Outcomes Measures

Outcome measures included (1) park use in terms of total numbers of park users, (2) total number of users by age and sex, (3) total number of users by physical activity (sitting or standing vs walking or vigorous activity), and (4) the mean level of physical activity among park users as measured by metabolic equivalent of task (MET) units. The total number of park users was operationalized as the mean number of users observed per scan at each park and at each wave. Similar means were computed for the number of users by age and sex. For park use by age, we combined children and teenagers into the youth group (20 years or younger) and adults and seniors into the adult group (21 years or older) to minimize the imprecision in age group identification inherent in park observations.

Physical activity was first determined by the number of park users engaging in different types of activities calculated as means across scans at each park for each wave. For this study, sitting and standing were combined into one category, and walking and vigorous activity (eg, jogging, running, bicycling, swimming, basketball, and other sports) were combined into another category (proxy of moderate-to-vigorous physical activity).

Finally, based on each park user’s physical activity level, energy expenditure estimates were calculated as MET units. Per the SOPARC protocol, we assigned 1.5 METs for sitting and standing, 3.0 METs for walking, and 6.0 METs for vigorous activities.^20^ Mean scores were calculated at the scan level.

### Statistical Analysis

We used independent samples *t* tests to compare park use and physical activity levels between the intervention and control sites at baseline. We used a difference-in-difference (DID) approach to determine the change in renovated park use compared with control park use. We explored separate models for total park use and park use by age group, sex, and physical activity level. The models included indicators for time (waves 1-3), intervention (renovation vs control parks), and time-by-intervention interaction. The analysis was performed at the scan level. Generalized estimation equations with negative binomial and normal distributions were used to account for correlated data among different scans and waves. We reported DID estimates comparing the magnitude of differential changes in intervention vs control parks on the relative rate ratio scale (DID RRR) and absolute difference scale (DID AD). We reported 95% CI and exact *P* values. Generalized estimation equations were also used to examine trends in park use and physical activity levels separately by intervention group. For sensitivity analysis, we examined potential COVID-19 effects by using data collected before 2020 only.

We used R, version 4.3.1 (R Project for Statistical Computing),^22^ for data cleaning and SPSS, version 28.0 (IBM Corp),^23^ for data analysis. We set the α value for statistical significance at 2-sided *P* < .05.

## Results

Across 3 waves of data collection, 28 322 park users were observed over 1458 scans in 54 parks. At baseline (wave 1), 6343 of 10 633 (59.7%) were youths 20 years or younger, 4927 of 10 632 (46.3%) were female, and 5705 (53.7%) were male. In addition, 4641 of 10 605 park users (43.8%) were sitting or standing. At baseline, control parks had more users than intervention parks (mean [SD], 23.1 [26.1] vs 17.0 [21.1] users/scan per park; *P* = .004). Sample characteristics overall and by intervention group are shown in Table 1.

**Table 1.** Baseline Park Use and Level of Physical Activity

Characteristic	All (N = 54)		Control (n = 21)		Intervention (n = 33)		*P* value^a^
	**No. of users/scan per park, mean (SD)**	**No. (%) of observations**	**No. of users/scan per park, mean (SD)**	**No. (%) of observations**	**No. of users/scan per park, mean (SD)**	**No. (%) of observations**	
	19.5 (23.4)	10 633 (100)	23.1 (26.1)	5132 (100)	17.0 (21.1)	5501 (100)	.004
Age group (n = 10 633)							
Youth (≤20 y)^b^	11.6 (16.3)	6343 (59.7)	14.1 (17.9)	3126 (60.9)	10.0 (14.8)	3217 (58.5)	.005
Adult (≥21 y)^c^	7.9 (9.8)	4290 (40.3)	9.0 (10.7)	2006 (39.1)	7.1 (9.0)	2284 (41.5)	.03
Sex (n = 10 632)							
Female	9.0 (13.0)	4927 (46.3)	11.4 (14.7)	2538 (49.5)	7.4 (11.4)	2389 (43.4)	<.001
Male	10.5 (12.2)	5705 (53.7)	11.7 (13.2)	2594 (50.5)	9.6 (11.5)	3111 (56.6)	.06
Level of physical activity (n = 10 605)							
Sitting or standing	8.5 (11.4)	4641 (43.8)	10.0 (12.9)	2214 (43.3)	7.5 (10.1)	2427 (44.2)	.02
Walking or vigorous physical activity	10.9 (14.5)	5964 (56.2)	13.1 (15.6)	2905 (56.7)	9.5 (13.5)	3059 (55.8)	.005
Metabolic equivalent for task units (n = 10 605)	3.7 (1.2)	NA	3.9 (1.1)	NA	3.6 (1.3)	NA	.02

Abbreviation: NA, not applicable.

^a^ Based on independent samples *t* tests.

^b^ Child and teenage park users were combined.

^c^ Adult and senior park users were combined.

### Total Park Use

Overall, the CPI was associated with a greater net number of park users at intervention vs control parks over time, and this association was sustained from wave 2 to wave 3 (wave 2 vs wave 1 DID RRR, 1.59 [95% CI, 1.16-2.18] users/scan [*P* = .004]; wave 3 vs wave 1 DID RRR, 1.69 [95% CI, 1.22-2.35] users/scan [*P* = .002]) (Table 2). Although the absolute number of park users increased at intervention parks over time, this change was not statistically significant (difference at wave 2, 3.75 [95% CI, −1.28 to 8.07] users/scan [*P* = .11]; difference at wave 3, 3.38 [95% CI, −0.84 to 8.57] users/scan [*P* = .16]). In contrast, park use decreased significantly in the control parks in the same time period (difference at wave 2, −4.98 [95% CI, −9.98 to −0.65] users/scan [*P* = .03]; difference at wave 3, −6.32 [95% CI, −11.10 to −2.62] users/scan [*P* = .002]) (Table 3 and Figure).

**Table 2.** DID Estimates of Intervention Association With Park Use and Level of Physical Activity Over Time

Time of measurement^a^	DID RRR estimate (95% CI)^b^	*P* value	DID AD estimate (95% CI)^c^	*P* value
**All park users**				
Wave 1	1 [Reference]	NA	1 [Reference]	NA
Wave 2	1.59 (1.16 to 2.18)	.004	9.18 (2.55 to 15.80)	.007
Wave 3	1.69 (1.22 to 2.35)	.002	10.25 (3.94 to 16.55)	.001
**Age group**				
Youth (≤20 y)^d^				
Wave 1	1 [Reference]	NA	1 [Reference]	NA
Wave 2	1.72 (1.19 to 2.50)	.004	6.18 (1.69 to 10.66)	.007
Wave 3	1.83 (1.26 to 2.66)	.002	6.68 (2.59 to 10.77)	.001
Adult (≥21 y)^e^				
Wave 1	1 [Reference]	NA	1 [Reference]	NA
Wave 2	1.42 (1.02 to 1.99)	.04	2.96 (0.01 to 5.91)	.049
Wave 3	1.53 (1.06 to 2.20)	.02	3.56 (0.54 to 6.58)	.02
**Sex**				
Female				
Wave 1	1 [Reference]	NA	1 [Reference]	NA
Wave 2	1.74 (1.23 to 2.44)	.002	5.12 (1.73 to 8.51)	.003
Wave 3	1.99 (1.33 to 2.99)	.001	6.28 (2.61 to 9.95)	.001
Male				
Wave 1	1 [Reference]	NA	1 [Reference]	NA
Wave 2	1.47 (1.03 to 2.09)	.03	4.05 (0.22 to 7.88)	.04
Wave 3	1.47 (1.04 to 2.06)	.03	3.95 (0.55 to 7.36)	.02
**Level of physical activity**				
Sitting or standing				
Wave 1	1 [Reference]	NA	1 [Reference]	NA
Wave 2	1.38 (0.97 to 1.98)	.08	2.90 (−0.69 to 6.48)	.11
Wave 3	1.51 (1.05 to 2.16)	.03	3.92 (0.15 to 7.68)	.04
Walking or vigorous physical activity				
Wave 1	1 [Reference]	NA	1 [Reference]	NA
Wave 2	1.84 (1.21 to 2.80)	.004	6.05 (1.45 to 10.65)	.01
Wave 3	2.01 (1.28 to 3.15)	.002	6.30 (2.05 to 10.56)	.004
**MET units**				
Wave 1	NA	NA	1	
Wave 2	NA	NA	0.48 (0.11 to 0.86)	.01
Wave 3	NA	NA	0.57 (0.22 to 0.93)	.002

Abbreviations: AD, absolute difference; DID, difference in difference; MET, metabolic equivalent of task; NA, not applicable; RRR, relative rate ratio.

^a^ Wave 1 indicates baseline, before renovation; wave 2, approximately 3 months post renovation or 2 years post baseline; and wave 3, 1 year post renovation or 3 years post baseline.

^b^ Represents the RRR based on generalized estimation equation (GEE) negative binomial regression.

^c^ Represents the AD based on GEE linear regression.

^d^ Child and teenage park users combined.

^e^ Adult and senior park users combined.

**Table 3.** Park Use and Level of Physical Activity in Intervention vs Control Parks Over Time

Time of measurement^a^	Control parks (n = 21)			Intervention parks (n = 33)		
	No. of users/scan per park, mean (SD)	Change vs wave 1 (95% CI)	*P* value^b^	No. of users/scan per park, mean (SD)	Change vs wave 1 (95% CI)	*P* value^b^
**Total park users**						
Wave 1	23.1 (26.1)	1 [Reference]	NA	17.0 (21.1)	1 [Reference]	NA
Wave 2	18.1 (18.8)	−4.98 (−9.98 to −0.65)	.03	20.8 (27.1)	3.75 (−1.28 to 8.07)	.11
Wave 3	16.8 (22.6)	−6.32 (−11.10 to −2.62)	.002	20.4 (25.4)	3.38 (−0.84 to 8.57)	.16
**Age group**						
Youth (≤20 y)^c^						
Wave 1	14.1 (17.9)	1 [Reference]	NA	10.0 (14.8)	1 [Reference]	NA
Wave 2	9.4 (12.5)	−4.67 (−8.41 to −1.46)	.005	11.1 (16.6)	1.17 (−1.59 to 4.08)	.39
Wave 3	8.6 (14.3)	−5.45 (−8.35 to −3.15)	<.001	10.9 (18.0)	0.89 (−2.22 to 4.09)	.56
Adult (≥21 y)^d^						
Wave 1	9.0 (10.7)	1 [Reference]	NA	7.1 (9.0)	1 [Reference]	NA
Wave 2	8.8 (9.6)	−0.27 (−1.97 to 1.31)	.70	9.7 (12.7)	2.60 (0.18 to 5.09)	.04
Wave 3	8.2 (10.1)	−0.87 (−3.28 to 1.08)	.32	9.6 (10.2)	2.49 (0.37 to 4.55)	.02
**Sex**						
Female						
Wave 1	11.4 (14.7)	1 [Reference]	NA	7.4 (11.4)	1 [Reference]	NA
Wave 2	8.6 (10.2)	−2.82 (−5.57 to −0.38)	.02	9.5 (13.3)	2.11 (−0.04 to 4.33)	.054
Wave 3	7.7 (12.2)	−3.77 (−6.37 to −1.72)	.001	9.7 (14.2)	2.29 (−0.59 to 5.09)	.12
Male						
Wave 1	11.7 (13.2)	1 [Reference]	NA	9.6 (11.5)	1 [Reference]	NA
Wave 2	9.5 (10.6)	−2.15 (−4.78 to 0.11)	.06	11.3 (15.1)	1.64 (−1.23 to 4.66)	.25
Wave 3	9.1 (11.8)	−2.54 (−5.25 to −0.38)	.02	10.7 (12.6)	1.08 (−1.25 to 3.54)	.35
**Level of physical activity**						
Sitting or standing						
Wave 1	10.0 (12.9)	1 [Reference]	NA	7.5 (10.1)	1 [Reference]	NA
Wave 2	10.8 (12.4)	0.78 (−1.58 to 2.84)	.58	11.0 (16.2)	3.49 (0.71 to 6.35)	.01
Wave 3	11.0 (15.0)	1.04 (−1.60 to 3.08)	.54	12.2 (17.1)	4.68 (1.71 to 7.62)	.002
Walking or vigorous physical activity						
Wave 1	13.1 (15.6)	1 [Reference]	NA	9.47 (13.51)	1 [Reference]	NA
Wave 2	7.4 (9.7)	−5.66 (−9.48 to −2.20)	.002	9.62 (15.13)	0.15 (−2.60 to 3.03)	.88
Wave 3	5.8 (9.8)	−7.30 (−10.80 to −4.26)	<.001	8.22 (10.83)	−1.25 (−3.95 to 1.50)	.38
**Mean MET**						
Wave 1	3.9 (1.1)	1 [Reference]	NA	3.6 (1.3)	1 [Reference]	NA
Wave 2	3.0 (1.1)	−0.88 (−1.13 to −0.62)	<.001	3.2 (1.2)	−0.39 (−0.67 to −0.12)	.005
Wave 3	2.5 (1.0)	−1.35 (−1.57 to −1.08)	<.001	2.9 (1.0)	−0.74 (−1.00 to −0.50)	<.001

Abbreviations: MET, metabolic equivalent of task; NA, not applicable.

^a^ Wave 1 indicates baseline, before renovation; wave 2, approximately 3 months post renovation or 2 years post baseline; and wave 3, 1 year post renovation or 3 years post baseline.

^b^ *P* values were based on generalized estimation equation linear regressions separately within the intervention and control parks.

^c^ Child and teen park users combined.

^d^ Adult and senior park users combined.

**Figure.**
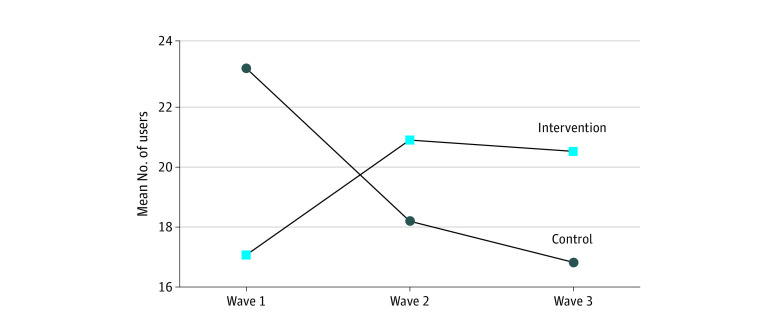
Park Use in Intervention vs Control Parks Over Time Changes within intervention parks were nonsignifcant (*P* ≥ .05); changes within control parks were significant (*P* < .05). Wave 1 indicates baseline, before renovation (reference point); wave 2, approximately 3 months post renovation or 2 years post baseline; and wave 3, 1 year post renovation or 3 years post baseline.

### Park Use by Age Group

Compared with control parks, the CPI boosted net park use over time among both youths (wave 2 vs wave 1 DID RRR, 1.72 [95% CI, 1.19-2.50] users/scan [*P* = .004]; wave 3 vs wave 1 DID RRR, 1.83 [95% CI, 1.26-2.66] users/scan [*P* = .002]) and adults (wave 2 vs wave 1 DID RRR, 1.42 [95% CI, 1.02-1.99] users/scan [*P* = .04]; wave 3 vs wave 1 DID RRR, 1.53 [95% CI, 1.06-2.20] users/scan [*P* = .02]) (Table 2). Among youths, park use remained steady over time in intervention parks while it decreased significantly in control parks (difference at wave 2, −4.67 [95% CI, −8.41 to −1.46] users/scan [*P* = .005]; difference at wave 3, −5.45 [95% CI, −8.35 to −3.15] users/scan [*P* < .001] ) (Table 3 and eFigure 2A in Supplement 1). Among adults, park use increased significantly at intervention parks (difference at wave 2, 2.60 [95% CI, 0.18-5.09] users/scan [*P* = .04]; difference at wave 3, 2.49 [95% CI, 0.37-4.55] users/scan [*P* = .02]) but remained unchanged at control parks (Table 3 and eFigure 2B in Supplement 1).

### Park Use by Sex

Compared with control parks, the CPI resulted in greater net overall park use over time for female users (wave 2 vs wave 1 DID RRR, 1.74 [95% CI, 1.23-2.44] users/scan [*P* = .002]; wave 3 vs wave 1 DID RRR, 1.99 [95% CI, 1.33-2.99] users/scan [*P* = .001]) and male users (wave 2 vs wave 1 DID RRR, 1.47 [95% CI, 1.03-2.09] users/scan [*P* = .03]; wave 3 vs wave 1 DID RRR, 1.47 [95% CI, 1.04-2.06] users/scan [*P* = .03]) (Table 2), due to a marginal though nonsignificant increase in female users at intervention parks and significant decreases of users of both sexes at control parks (Table 3 and eFigure 2C-D in Supplement 1).

### Physical Activity

The CPI led to a net positive increase of users engaged in sitting or standing at intervention vs control parks over time (wave 3 vs wave 1 DID RRR, 1.51 [95% CI, 1.05-2.16] users/scan; *P* = .03) (Table 2). Sitting or standing increased significantly at intervention parks (difference at wave 2, 3.49 [95% CI, 0.71-6.35] users/scan [*P* = .01]; difference at wave 3, 4.68 [95% CI, 1.71-7.62] users/scan [*P* = .002]) but did not change at control parks (Table 3 and eFigure 3A in Supplement 1).

Furthermore, there was a net positive number of users engaged in walking or vigorous physical activity at intervention vs control parks over time (wave 2 vs wave 1 DID RRR, 1.84 [95% CI, 1.21-2.80] users/scan [*P* = .004]; wave 3 vs wave 1 DID RRR, 2.01 [95% CI, 1.28-3.15] users/scan [*P* = .002]) (Table 2). Park users engaging in walking or vigorous physical activity did not change significantly at intervention parks but decreased significantly at control parks (difference at wave 2, −5.66 [95% CI, −9.48 to −2.20] users/scan [*P* = .002]; difference at wave 3, −7.30 [95% CI, −10.80 to −4.26] users/scan [*P* < .001]) (Table 3 and eFigure 3B in Supplement 1).

### Association With MET

After the CPI renovation, a net positive change in MET in intervention vs control parks occurred over time (wave 2 vs wave 1 DID AD, 0.48 [95% CI, 0.11-0.86] U/scan [*P* = .01]; wave 3 vs wave 1 DID AD, 0.57 [95% CI, 0.22-0.93] U/scan [*P* = .002]) (Table 2). The change was due to a greater decrease in MET at control vs intervention parks (Table 3 and eFigure 3C in Supplement 1).

### Sensitivity Analysis

To examine the effect of COVID-19, we performed the same analysis using data collected before 2020 only. Results (eTable in Supplement 1) mirrored those of the full sample.

## Discussion

To our knowledge, this is one of the first large-scale quality improvement studies to evaluate the association of citywide park redesign and renovation with park use, leveraging a major municipal initiative as a natural experiment. The CPI was associated with a net positive effect on park use at intervention vs control parks, both immediately post renovation and at the second follow-up. This was mainly due to a significant increase in park use among adults at intervention sites and an overall decrease in park use at control sites. The CPI also was associated with a net increase of both lower and higher physical activity among park users at intervention vs control sites. However, MET output decreased in both groups, with a smaller decline at intervention than control sites.

The CPI entailed various microenvironmental enhancements, including aesthetic improvements, age-friendly and gender-neutral designs, and accommodations for different physical activity levels. Parks introduced customized exercise spaces based on community preferences, such as multipurpose fields, playgrounds, fitness equipment, and sports courts. Additionally, increases in shading, restrooms, drinking fountains, seating, and accessibility features were incorporated. Enhanced security lighting was added for nighttime safety. Adult- and senior-friendly features likely contributed to increased park use among these age groups. Many parks previously catered to children as playgrounds.

Our findings support those of prior research showing that park renovations could have a robust positive impact on park use.^7^ However, our findings stand in contrast to another study reporting no change in park use at 1-year follow-up in low-income neighborhoods.^12^ Community engagement by NYC Parks likely aligned local preferences with renovation features, enhancing their impact. Still, our nuanced results partly stemmed from significant decreases in park use at control sites. While a shift in users from control to intervention sites is possible, given that study parks were at least 0.6 miles apart, this scenario was likely minimized.

In a review of 24 studies,^11^ male individuals typically used parks more than female individuals (as was the case at our study baseline), but our study indicated that the CPI resulted in a more pronounced net increase in female users. While the increase was of borderline significance, it suggests the CPI’s potential for more equitable park use between sexes. A study across 162 neighborhood parks in 25 US cities highlighted the lack of quality parks in proximity (eg, poor security) as a major barrier for female users.^24^ The CPI’s safety-enhancing renovations possibly contributed to attracting more female users to intervention parks. Past research also suggests that more women, compared with men, take children to parks and engage in sitting or standing activities, such as socializing with friends and family.^25^ The CPI increased seating and shading in parks.

Although the CPI incorporated exercise spaces and equipment for adults, we found an overall decrease in physical activity in both control and intervention parks, albeit more pronounced in the former. Prior studies^7,26,27^ indicated mixed results regarding the impact of park renovation on physical activity, suggesting that physical enhancements alone may not suffice to bolster physical activity levels. For example, a study in a suburban community outside Denver, Colorado, also found that park renovation increased park use but not physical activity.^28^ Our own prior research also underscored that the social environment of parks (eg, social and cultural events), beyond physical attributes, drives park use.^29^ This perspective might extend to physical activity–focused programming within parks. Factors such as elevated stress levels and chronic health disparities in low-income communities impede park-based physical activity.^30,31^ Hence, a comprehensive approach seems crucial to maximize park use for physical activity.

### Strengths and Limitations

Strengths of this study include a prospective study design in a large sample of parks, standardized and observer-assessed park use, and use of matched controls, all limitations in prior studies.^32^ Nonetheless, our study has some limitations. First, although data collectors were trained thoroughly, there is a possibility of observer bias (eg, in identifying age groups) or bias due to lack of blinding to park group (renovation vs control parks). Second, due to the snapshot nature of audits, physical activity levels may not have been captured accurately. For example, a person may be running but counted as sitting or standing since they stopped to rest during the audit. Third, most observations were conducted during summer, and park use patterns may differ during other seasons.^33^ Fourth, although we found the same results in pre–COVID-19 data, we could not completely rule out the possibility of COVID-19 suppressing park use or physical activity in both groups. Fifth, while not uncommon when assessing large real-world interventions, the DID analysis included only 1 round of prerenovation data collection, thus limiting the ability to assess the parallel trends assumption underlying such analysis. However, given that the study involved parks densely distributed in a single city and the relatively large number of parks in the intervention and control groups, we do not have any plausible reason to believe that significant differential changes (increase or decrease) in park use would have occurred between the intervention and control parks prior to the onset of the CPI. Last, study power could have limited our ability to detect statistical significance in some of the analyses.

## Conclusions

In this quality improvement study, park redesign and renovation were associated with increased park use in low-income neighborhoods, offering a rare opportunity to evaluate initiatives on park use and park-based physical activity in predominantly Black and Latino communities. The large number of park sites and longitudinal design with matched controls are major strengths in this study. While our findings suggest a positive impact on park use, especially among adults, more research is needed for understanding park-related physical activity. This study may inform future urban development and public health policies regarding parks.

## Notes

Supplement 1. **eFigure 1.** Timeline of Data Collection Across Waves and Years at Intervention (Top) and Control (Bottom) Parks **eFigure 2.** Park Use by Age Group and Sex in Intervention vs Control Parks Over Time **eFigure 3.** Park Use by Level of Physical Activity in Intervention vs Control Parks Over Time **eTable.** Difference-in-Difference Estimates of Intervention Effects on Park Use and Level of Physical Activity Over Time, 2016 to 2019 (Pre–COVID-19 Pandemic Period)

Supplement 2.
Data Sharing Statement
