# Supplementary material for: Association of Park Renovation With Park Use in New York City
Source: JAMA Netw Open. 2024 Apr 10;7(4):e241429. doi: 10.1001/jamanetworkopen.2024.1429 (PMC11007573; doi:10.1001/jamanetworkopen.2024.1429)
Supplement: Supplement 1. — eFigure 1. Timeline of Data Collection Across Waves and Years at Intervention (Top) and Control (Bottom) Parks eFigure 2. Park Use by Age Group and Sex in Intervention vs Control Parks Over Time eFigure 3. Park Use by Level of Physical Activity in Intervention vs Control Parks Over Time eTable. Difference-in-Difference Estimates of Intervention Effects on Park Use and Level of Physical Activity Over Time, 2016 to 2019 (Pre–COVID-19 Pandemic Period) [file jamanetwopen-e241429-s001.pdf]

Supplementary Online Content

Kodali HP, Wyka EK, Costa SA, Evenson KR, Thorpe LE, Huang TTK. Association of park renovation with park use in New York City. *JAMA Netw Open*. 2024;7(4):e241429. doi:10.1001/jamanetworkopen.2024.1429

eFigure 1. Timeline of Data Collection Across Waves and Years at Intervention (Top) and Control (Bottom) Parks

eFigure 2. Park Use by Age Group and Sex in Intervention vs Control Parks Over Time

eFigure 3. Park Use by Level of Physical Activity in Intervention vs Control Parks Over Time

eTable. Difference-in-Difference Estimates of Intervention Effects on Park Use and Level of Physical Activity Over Time, 2016 to 2019 (Pre–COVID-19 Pandemic Period)

This supplementary material has been provided by the authors to give readers additional information about their work.

© 2024 Kodali HP et al. *JAMA Network Open*.

eFigure 1. Timeline of Data Collection Across Waves and Years at Intervention (Top) and Control (Bottom) Parks

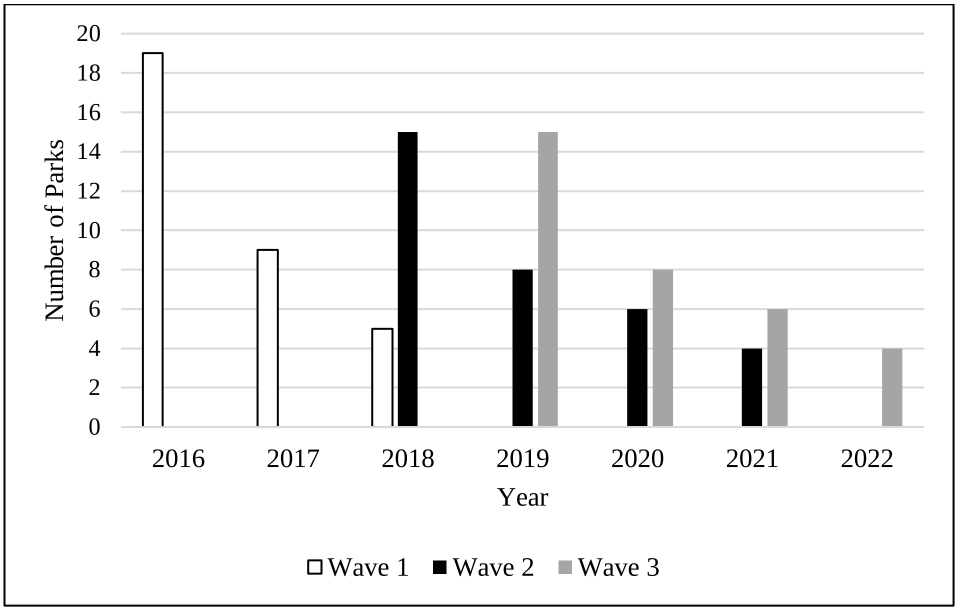

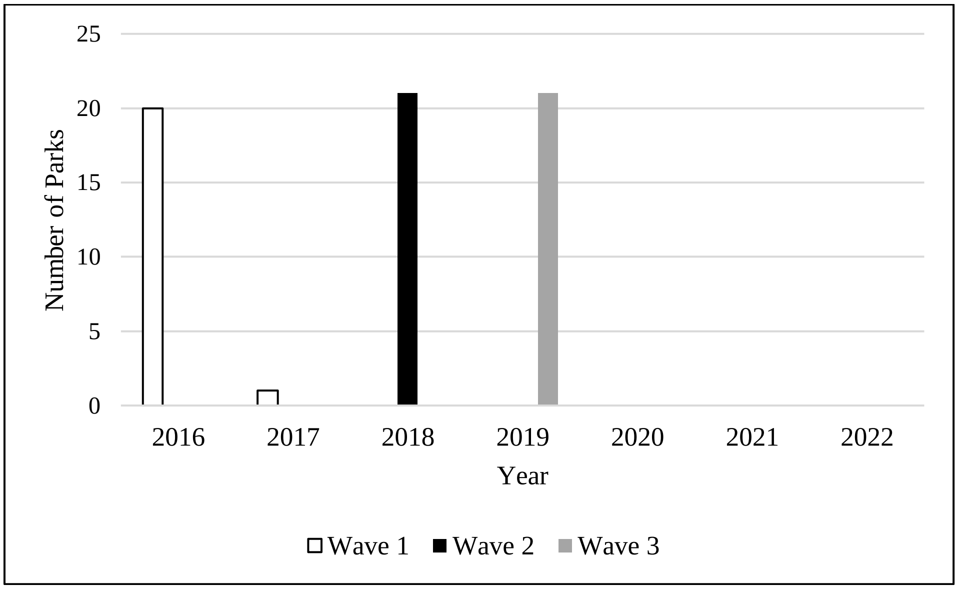

Note. Wave 1 = baseline, pre-renovation (reference); Wave 2 = approximately 3 months post-renovation or 2 years post-baseline; Wave 3 = 1-year post-renovation or 3 years post-baseline.

© 2024 Kodali HP et al. *JAMA Network Open*.

eFigure 2. Park Use by Age Group and Sex in Intervention vs Control Parks Over Time

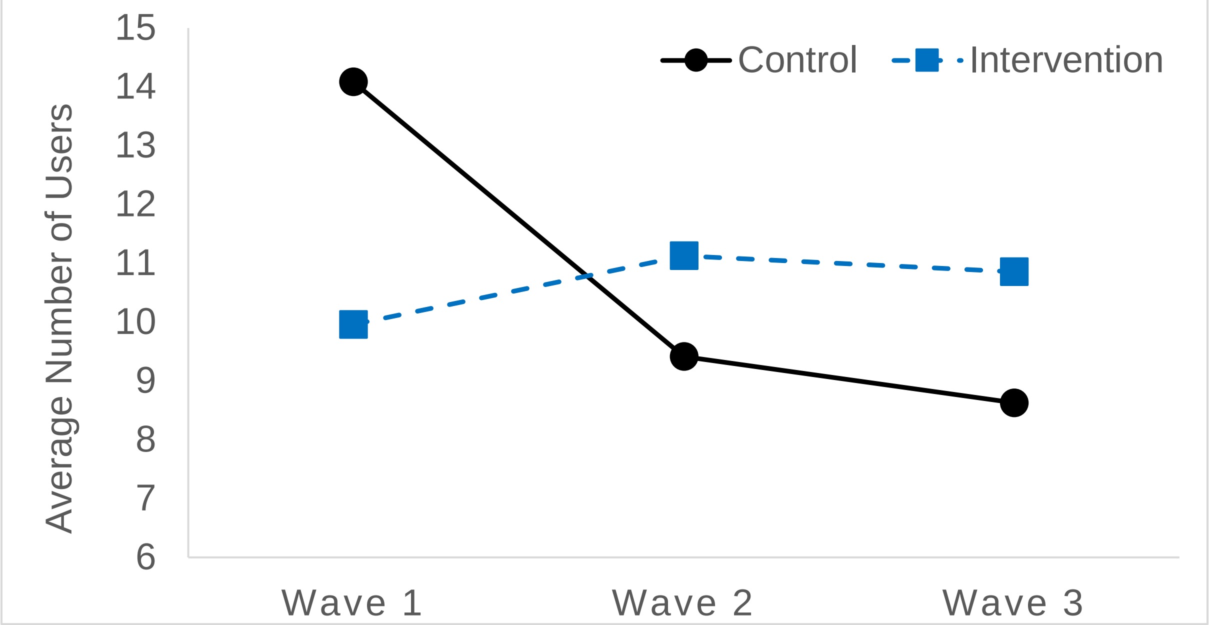

Panel 2a. Park Usage among Youth (≤20 y)

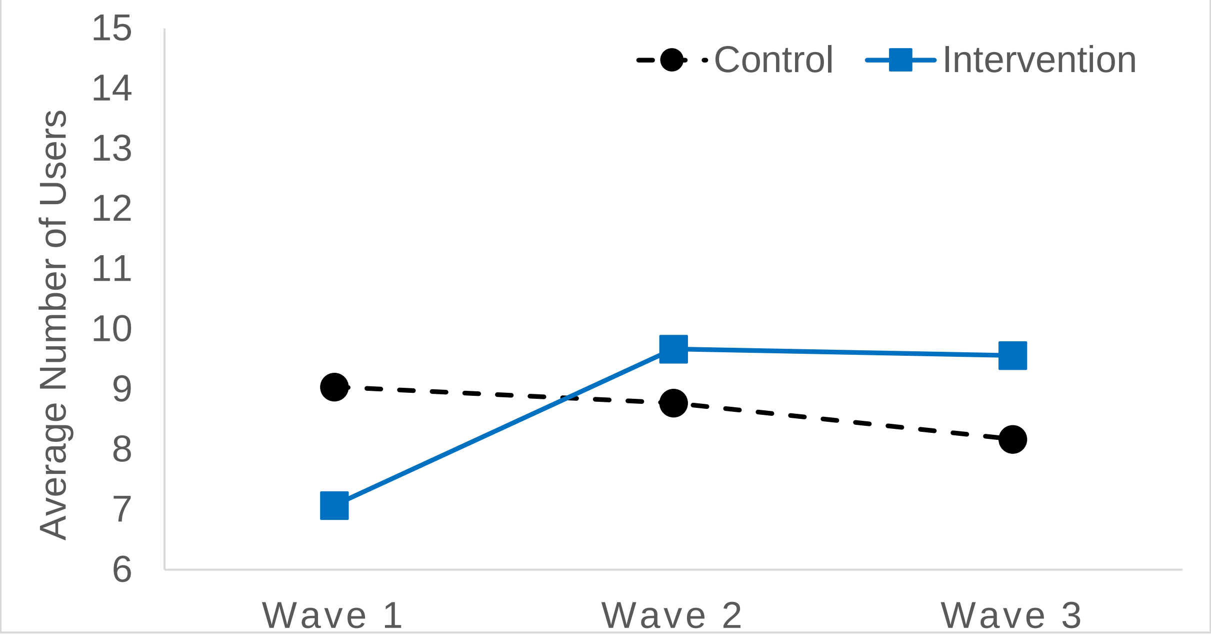

Panel 2b. Park Usage among Adults (≥21 y)

© 2024 Kodali HP et al. *JAMA Network Open*.

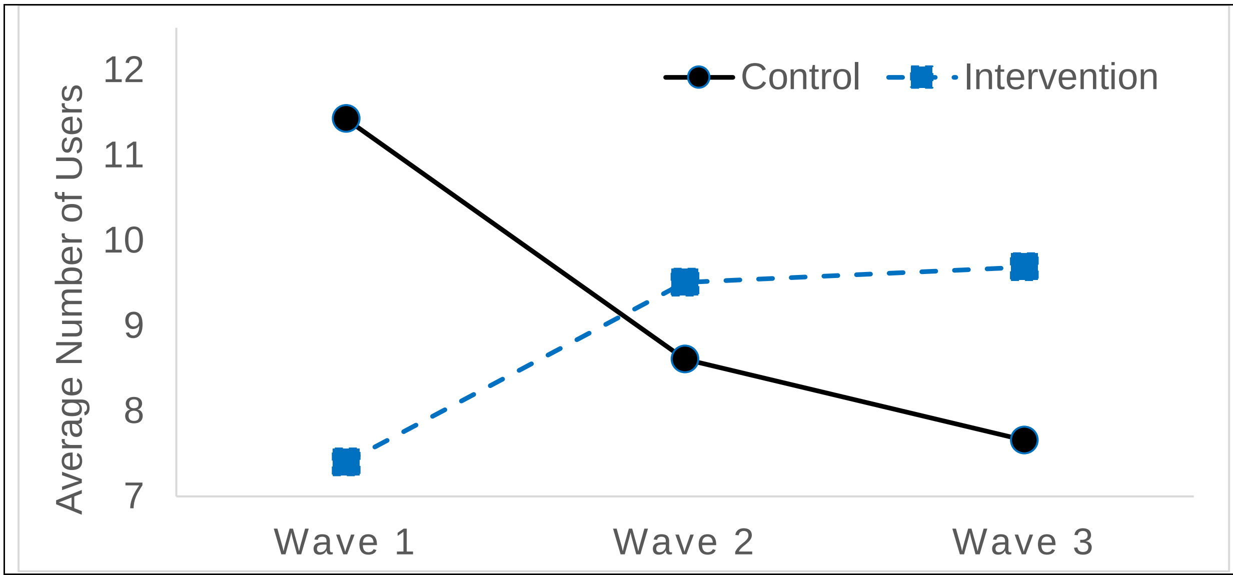

Panel 2c. Park Usage among Females

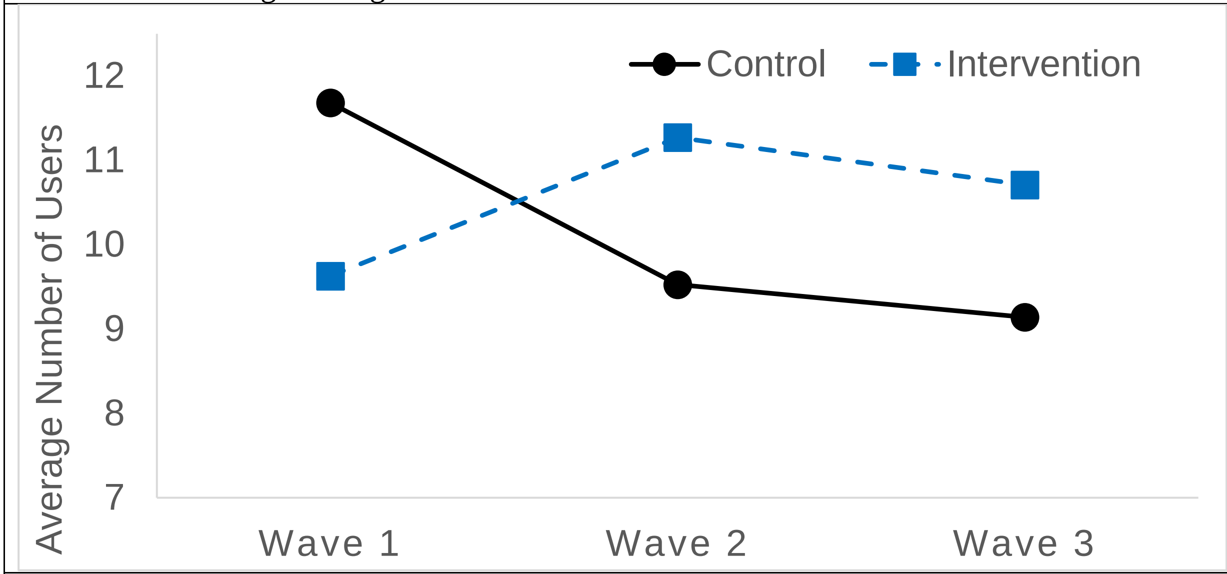

Panel 2d. Park Usage among Males

Note. Solid lines indicate significant ($P<0.05$) and dashed lines non-significant changes within intervention or control parks. Wave 1 = baseline, pre-renovation (reference); Wave 2 = approximately 3 months post-renovation or 2 years post-baseline; Wave 3 = 1-year post-renovation or 3 years post-baseline.

© 2024 Kodali HP et al. *JAMA Network Open*.

eFigure 3. Park Use by Level of Physical Activity in Intervention vs Control Parks Over Time

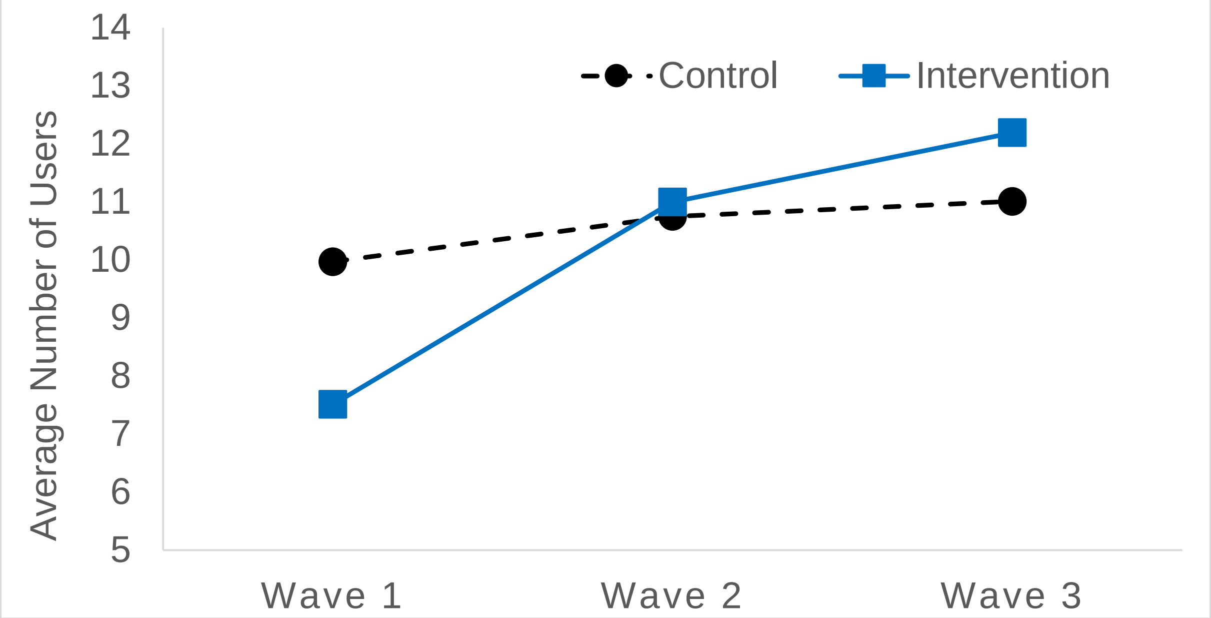

Panel 3a. Park Usage for Sitting/Standing

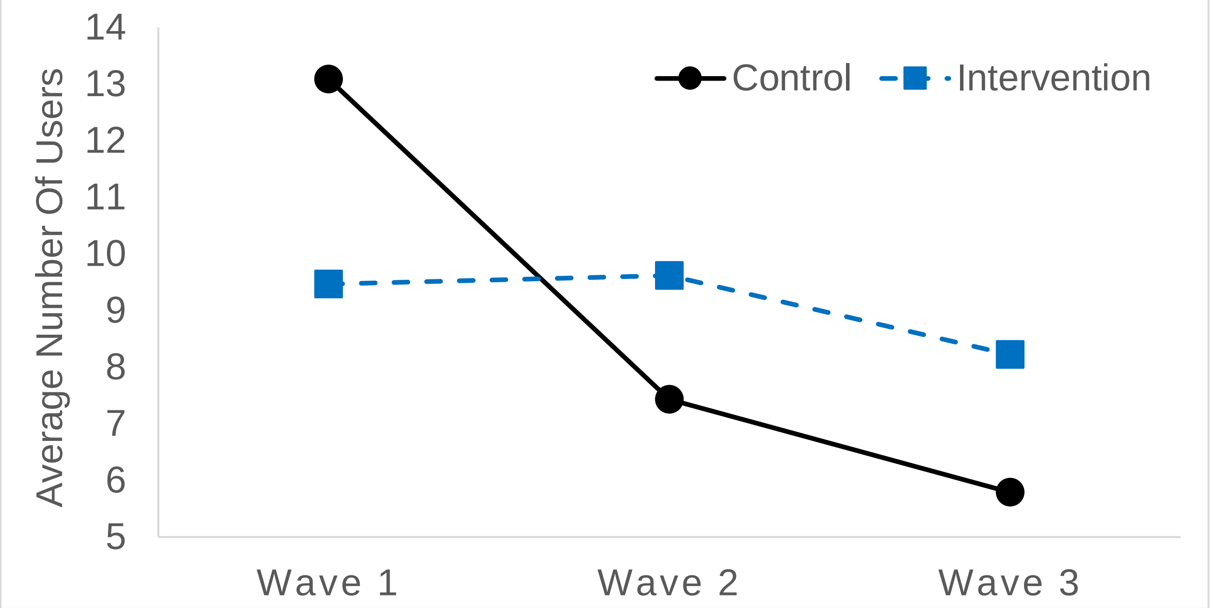

Panel 3b. Park Usage for Walking/Vigorous PA

© 2024 Kodali HP et al. *JAMA Network Open*.

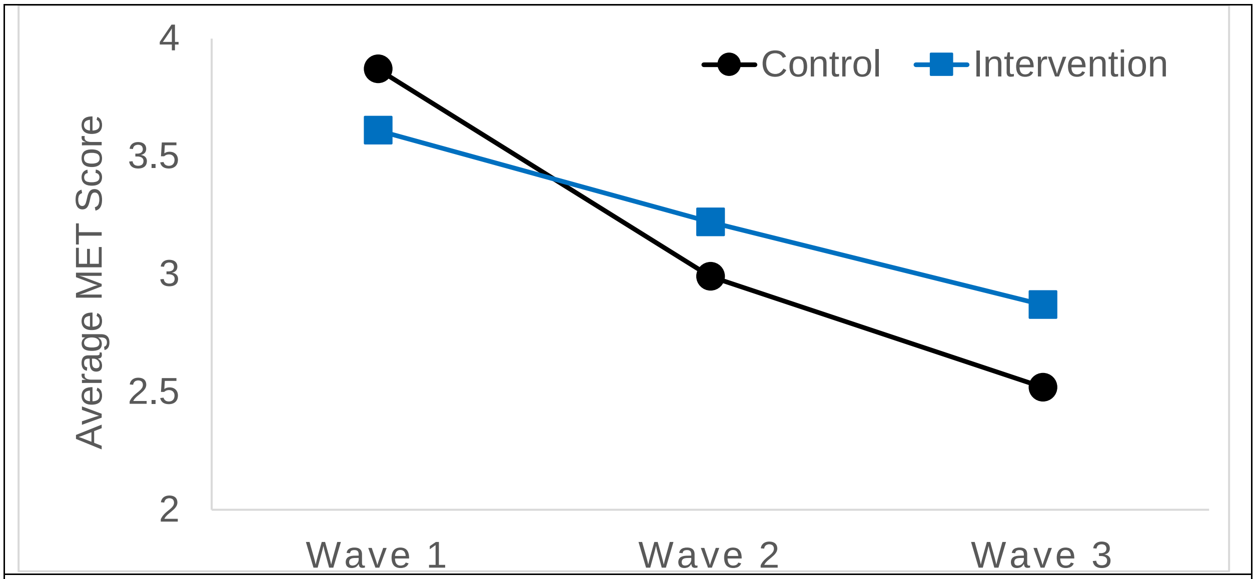

Panel 3c. Change in Metabolic Equivalent for Task (MET)

Note. Solid lines indicate significant (P<0.05) and dashed lines non-significant changes within intervention or control parks. Wave 1 = baseline, pre-renovation (reference); Wave 2 = approximately 3 months post-renovation or 2 years post-baseline; Wave 3 = 1-year post-renovation or 3 years post-baseline.

© 2024 Kodali HP et al. *JAMA Network Open*.

eTable. Difference-in-Difference Estimates of Intervention Effects on Park Use and Level of Physical Activity Over Time, 2016 to 2019 (Pre–COVID-19 Pandemic Period)

	**DID_{RRR} estimate [c] (95% CI)**	**P value**	**DID_{AD} estimate [d] (95% CI)**	**P value**
**Total Park Users**				
Wave 1	1		1	
Wave 2	1.61 (1.17, 2.23)	0.004	9.46 (2.73, 16.19)	0.006
Wave 3	2.00 (1.38, 2.90)	< 0.001	13.37 (5.74, 20.99)	0.001
**Age Group**				
Youth (≤20 y) users [a]				
Wave 1	1		1	
Wave 2	1.83 (1.25, 2.67)	0.002	6.85 (2.27, 11.44)	0.003
Wave 3	2.45 (1.64, 3.66)	< 0.001	10.05 (5.00, 15.1)	<.001
Adult (≥21 y) users [b]				
Wave 1	1		1	
Wave 2	1.37 (0.97, 1.94)	0.078	2.57 (-0.43, 5.57)	0.093
Wave 3	1.52 (1.03, 2.24)	0.036	3.27 (0.02, 6.52)	0.049
**Gender**				
Female users				
Wave 1	1		1	
Wave 2	1.75 (1.23, 2.49)	0.002	5.19 (1.75, 8.64)	0.003
Wave 3	2.36 (1.52, 3.67)	< 0.001	7.69 (3.31, 12.07)	0.001
Male users				
Wave 1	1		1	
Wave 2	1.50 (1.04, 2.16)	0.029	4.25 (0.27, 8.23)	0.036
Wave 3	1.73 (1.20, 2.51)	0.004	5.65 (1.67, 9.63)	0.005
**Level of Physical Activity (PA)**				
Users engaged in sitting/standing				
Wave 1	1		1	
Wave 2	1.57 (1.11, 2.23)	0.011	4.04 (0.20, 7.88)	0.039
Wave 3	2.10 (1.52, 2.89)	< 0.001	7.00 (2.12, 11.88)	0.005
Users engaged in walking/vigorous PA				
Wave 1	1		1	
Wave 2	1.71 (1.13, 2.59)	0.012	5.34 (0.80, 9.88)	0.021
Wave 3	2.01 (1.16, 3.47)	0.013	6.28 (1.16, 11.41)	0.016
**Metabolic Equivalent of Task (MET)Units**				
Wave 1	NA	NA	1	
Wave 2	NA	NA	0.37 (-0.03, 0.77)	0.068
Wave 3	NA	NA	0.10 (-0.20, 0.41)	0.517

Note. Wave 1 = baseline, pre-renovation (reference); Wave 2 = approximately 3 months post-renovation or 2 years post-baseline; Wave 3 = 1-year post-renovation or 3 years post-baseline. [a] child and teen park users combined, [b] adult and senior park users combined. [c] DID_{RRR} estimate - difference-in-difference estimate represents the relative rate ratio based on generalized estimation equation (GEE) negative binomial regression, [d] DID_{AD} estimate - difference-in-difference estimate represents absolute difference based on GEE linear regression. Abbrevaition: CI - Confidence Interval.

© 2024 Kodali HP et al. *JAMA Network Open*.
