# Supplementary material for: Association of Park Renovation With Park Use in New York City
Source: JAMA Netw Open. 2024 Apr 10;7(4):e241429. doi: 10.1001/jamanetworkopen.2024.1429 (PMC11007573; doi:10.1001/jamanetworkopen.2024.1429)
Supplement: Supplement 2. — Data Sharing Statement [file jamanetwopen-e241429-s002.pdf]

Data Sharing Statement

Kodali. Association of Park Renovation With Park Use in New York City. *JAMA Netw Open*. Published April 10, 2024. doi:10.1001/jamanetworkopen.2024.1429

Data
Data available: Yes
Data types: Deidentified participant data, Data dictionary
How to access data: Requests to access the datasets should be directed to terry.huang@sph.cuny.edu.
When available: beginning date: 01-01-2024

Supporting Documents
Document types: None

Additional Information
Who can access the data: the data will be available from the corresponding author on reasonable request.
Types of analyses: for non-commercial research purposes only
Mechanisms of data availability: A part of the data belongs to NYC Parks which needs to approve data requests
